# Progress and Challenges in Transfer of Large‐Area Graphene Films

**DOI:** 10.1002/advs.201500343

**Published:** 2016-02-04

**Authors:** Yi Chen, Xiao‐Lei Gong, Jing‐Gang Gai

**Affiliations:** ^1^ State Key Laboratory of Polymer Materials EngineeringPolymer Research Institute of Sichuan UniversityChengduSichuan610065China

**Keywords:** challenges, dry transfer methods, graphene films, promising methods, wet transfer methods

## Abstract

Graphene, the thinnest, strongest, and stiffest material with exceptional thermal conductivity and electron mobility, has increasingly received world‐wide attention in the past few years. These unique properties may lead to novel or improved technologies to address the pressing global challenges in many applications including transparent conducting electrodes, field effect transistors, flexible touch screen, single‐molecule gas detection, desalination, DNA sequencing, osmotic energy production, etc. To realize these applications, it is necessary to transfer graphene films from growth substrate to target substrate with large‐area, clean, and low defect surface, which are crucial to the performances of large‐area graphene devices. This critical review assesses the recent development in transferring large‐area graphene grown on Fe, Ru, Co, Ir, Ni, Pt, Au, Cu, and some nonmetal substrates by using various synthesized methods. Among them, the transfers of the most attention kinds of graphene synthesized on Cu and SiC substrates are discussed emphatically. The advances and the main challenges of each wet and dry transfer method for obtaining the transferred graphene film with large‐area, clean, and low defect surface are also reviewed. Finally, the article concludes the most promising methods and the further prospects of graphene transfer.

## 1 Introduction

This is an open access article under the terms of the Creative Commons Attribution License, which permits use, distribution and reproduction in any medium, provided the original work is properly cited.

As an exotic 2D material composed of sp^2^ hybrid carbon atoms, graphene has attracted growing attention since it was isolated by both Geim and Novoselov from graphite by mechanical exfoliation.^1^ Graphene has demonstrated a variety of unique properties such as superior mechanical properties with Young's modulus of 1 TPa,^2^ high electrical conductivity at room temperature (200 000 cm^2^ V^−1^ s^−1^)^3^ and exceptional thermal conductivity (5000 W m^−1^ K^−1^).^4^ These intriguing properties enable large‐area graphene (LAG) with controllable thickness to have many potential applications including field‐effect transistors (FETs),^5^, ^6^ supercapacitor,^7^, ^8^, ^9^ electrochemical sensing,^10^, ^11^ solar cells,^12^, ^13^ wearable electronics devices,^14^ display devices for OLEDs,^15^ and touch‐screen,^16^ highly selective molecular sieve membranes for single‐molecule gas detection,^17^ desalination,^18^, ^19^ and DNA sequencing.^20^ To realize these applications in large scale, the first challenge is to synthesize LAG with excellent properties.

Recently, many methods have been used to synthesize graphene, such as graphite oxide reduction method,^21^, ^22^ chemical vapor deposition method (CVD),^23^, ^24^ and epitaxial growth method.^25^, ^26^ Among these methods, both epitaxial growth of graphene on the SiC surface and CVD on the transition metals are efficient methods to produce LAG. Especially, CVD method is considered to be the most efficient method in industrial application, for its low cost, high quality and large area.^27^ It was reported that 30 in. LAG film was synthesized through CVD method by Samsung in 2010.^28^

The growth substrates for synthesizing LAG film have great influence on the properties of graphene. Besides, the methods of directly synthesizing graphene on target substrates are very immature now. So, the LAG films synthesized by both CVD method and the epitaxial growth method through SiC have to be transferred from the growth substrates to the target substrates in the applications of LAG films. Up to now, various methods have been developed to transfer the graphene film, many of which are of advantages for industrial applications. No doubt the development of the transfer methods will promote the study and application of LAG films. This work aims to give a comprehensive review of the method for transferring graphene from various growth substrates to the target substrates.

In this review, we first discuss the transfer methods of graphene grown on different kinds of metal substrates and focus on the transfer of graphene grown on Cu substrate. Then, the progress in each wet or dry transfer method is reviewed along with the properties of the transferred graphene film, respectively. In the third part, the transfer of epitaxial graphene grown on SiC is discussed. The properties of the transferred graphene film by different methods are listed in **Table** **1**. Finally, the research activities and the possible future research directions are summarized, with the main challenges in the transfer of graphene as the highlight. In the meanwhile, the most available transfer method for industrial application is also proposed.

**Table 1:** The properties of the transferred graphene film by different methods

Method	Growth substrate	Target substrate	[G cm^−1^]	[2D cm^−1^]	[D cm^−1^]	*I*_2D_/*I*_G_	*I*_D_/*I*_G_	Sheet resistance	Field‐effect mobility	No. layer	Refs.
Wet transfer	Cu	SiO_2_/Si	1560–1620	2660–2700	1300–1400	2.0			4050 cm^2^ v^−1^ s^−1^ (carrier‐mobility)	95% one‐layer	23
(polymer‐assisted)	Cu	SiO_2_/Si glass	1588	2690	1350	8.0–11.0	0.06–0.12	980 Ω sq^−1^		one‐layer	43
	Cu	SiO_2_/Si PET	1583	2642	1320	3.1	0.06	255 Ω sq^−1^ (on glass)	5602 cm^2^ v^−1^ s^−1^ (hole‐mobility)	one‐layer	56
									4535 cm^2^ v^−1^ s^−1^ (electron mobility)		
Polymer‐free	Cu	SiO_2_/Si		2700				810 Ω sq^−1^	6300 cm^2^ v^−1^ s^−1^ (on BN)	One‐layer	54
Electrochemical	Pt	SiO_2_/Si	1590	2685		2.0–3.0	<0.05		7100 cm^2^ v^−1^ s^−1^	Mostly one‐layer	60
	Cu	PET	1582	2678				275 Ω sq^−1^	350 cm^2^ v^−1^ s^−1^ (6 K)	>95% one‐layer	28
Dry transfer	Cu	PDMS	1584	2681	1350	2.43	0.2			one‐layer	76
	SiC	SiO_2_/Si	1530	2700	1380		0.037 ± 0.008	175 Ω sq^−1^	1348 cm^2^ v^−1^ s^−1^	one‐layer	80
Face‐to‐face	Cu	SiO_2_/Si	1560–1620	2690	1350				3800 cm^2^ v^−1^ s^−1^ (hole mobility)	one‐layer	36
Transfer free	SiO_2_/Si		1592	2685	1351	>2			667 cm^2^ v^−1^ s^−1^ (room temperature)	one‐layer	89

Note: 2D/G ration indicates the doping level of graphene film. The high *I*_2D_/*I*_G_ indicates a lower doping level of graphene. D/G ration indicates the quality of graphene film. The low *I*_D_/*I*_G_ indicates a higher quality of graphene film.

## 2 Transfer of Graphene Synthesized by CVD Method on Metal Substrates

Chemical vapor deposition method is believed to be an efficient method to synthesize LAG. The mechanism of CVD method is described as follows: decompose the hydrocarbon precursors (CH_4_) at high temperature and then deposit the decomposition products on metal substrate to form single‐layer or few‐layers graphene. Graphene synthesized by CVD method has been realized on many metal substrates, such as Fe,^29^ Ru,^30^ Co,^31^ Ir,^32^ Ni,^24^ Pt,^33^ Au,^34^ and Cu.^23^, ^35^ To transfer graphene grown on these metal substrates, etching of the metals is the efficient and straightforward way. Fe, Ru, Co, Ni, and Cu can be etched by their etchants easily. But, for the chemically insert or noble metal substrates, such as Ir, Pt, and Au, the traditional wetting transfer methods are not available, because these metals are difficult to be etched away completely or the cost is too high. The electrochemical delamination method, which will be discussed later seems to be an efficient way to transfer graphene grown on these metal substrates.

An et al.^29^ successfully synthesized graphene on Fe substrate. The graphene was transferred by using polymethyl methacrylate (PMMA) as the supporting layer and HCl solution as the Fe etchant. But the thick graphene films were more likely to get broken, especially during the step of PMMA removal. For graphene growing on Ni substrate, Kim et al.^24^ used the FeCl_3_ solution as the Ni etchant. The graphene film was separated from the Ni substrate in a few minutes, and then it was transferred to arbitrary substrate. They also developed a dry‐transfer process by using polydimethylsiloxane (PDMS) stamp as the supporting layer. Various sizes of graphene film can be transferred to arbitrary substrate. For graphene growing on Co film, Wang et al.^31^ used the FeCl_3_ solution as the Co etchant. After the removal of Co film, the graphene film was transferred to the target substrates.

Among all of these methods for growth graphene, CVD synthesis of graphene on Cu foil is of many advantages. For example, the C solubility of Cu is low, the graphene layers can be controlled easily, and it can also be transferred easily with relatively low cost. Therefore, Cu has been often used as the metal substrate. Here, we mainly focus on the transfer of graphene synthesized on Cu substrate. The transfer methods for CVD graphene can be classified as wet and dry transfer, depending on the environment where graphene touches the target substrate.^36^

### 2.1 Wet Transfer Method

#### 2.1.1 Wet Transfer Method and Supporting Layer

Generally, taking advantage of the polymer layer such as PMMA as a supporting layer is a necessary step for wet method of transferring graphene grown by CVD. PMMA has many prominent features, such as the relatively low viscosity, excellent wetting capability, flexibility, and good dissolubility in several organic solvents.^37^ Most importantly, the high transparency of the PMMA makes it clear to observe the process of Cu removal. The usual steps are described as follows: (1) in the CVD method, graphene is grown on both sides of the Cu foil, so one side of the graphene on Cu substrate is first removed away by the O_2_ plasma in order to etch the Cu substrate; 2) PMMA layer is coated on the surface of the graphene/copper substrate and then cured; 3) the underlying copper substrate is etched away by the etchant, then washed by the deionized (DI) water; 4) transfer the PMMA/graphene sample to the target substrate; 5) the PMMA layer is removed by acetone, then washed by the DI water and dry.

Besides using PMMA as the polymer supporting layer, poly(bisphenol A carbonate) (PC)^38^ is another kind of polymer used in the wet‐transfer method. Lin et al. replaced the PMMA with PC for transferring the graphene. Compared with PMMA, PC can be removed easily by organic solvents (chloroform) without the further annealing to realize clean transfer. The transfer process is similar to the method using the PMMA as the supporting layer. Polymer polyisobutylene (PIB) has also been used as the supporting layer in the wet‐transfer method. Song et al.^39^ used the PIB as the self‐release layer. It was inserted between the PDMS stamp and the graphene sheet. The graphene was then transferred in two ways. For the dry transfer method, PDMS stamp was removed first then PIB was removed by decane, hexane, or squalane on the spinner for three cycles. For the wet transfer method, PDMS was still kept conformal contact with the PIB. The removal solvent (decane) was flooded over the target substrate, and soaked for 5 min to remove the PIB layer. The transferred graphene surface can be molecularly clean. But in most cases, PMMA was used as the polymer supporting layer.

#### 2.1.2 Transferring Graphene Without Degraded Quality

**Figure 1:**
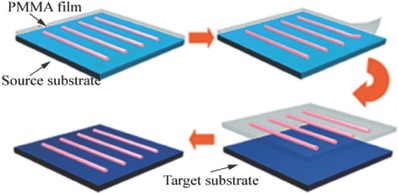
Illustration of the process of PMMA‐mediated nanotransfer printing technique. Reproduced with permission.^37^ 2008, American Chemical Society.

The transfer of carbon nanotubes (**Figure** **1**)^37^ has inspired the first attempt of using the PMMA mediator to transfer the graphene from SiO_2_/Si substrate to SiC substrate.^40^ Almost no morphology change or significant fold has been induced by the transfer process. The “PMMA‐mediator” was used to transfer the graphene film synthesized on Cu substrate in 2009.^23^

Li et al.^41^ found that graphene transferred by this method was easy to form cracks during the transfer process. They attributed this phenomenon to the fact that the graphene had not been fully contacted with the target substrate, so the cracks were formed when the PMMA layer was removed away. The Cu substrate undergoes surface reconstruction at high temperature, which is attributed to the rough metal surface. Graphene followed the surface of the underlying metal substrate, so the surface of the graphene was rough when the Cu was etched away. Thus, gaps were formed between the graphene and the target substrate. These gaps tended to crack during PMMA removal. So graphene films were easy to form cracks. The graphene must be fully contacted with the target substrate during the transfer process.

**Figure 2:**
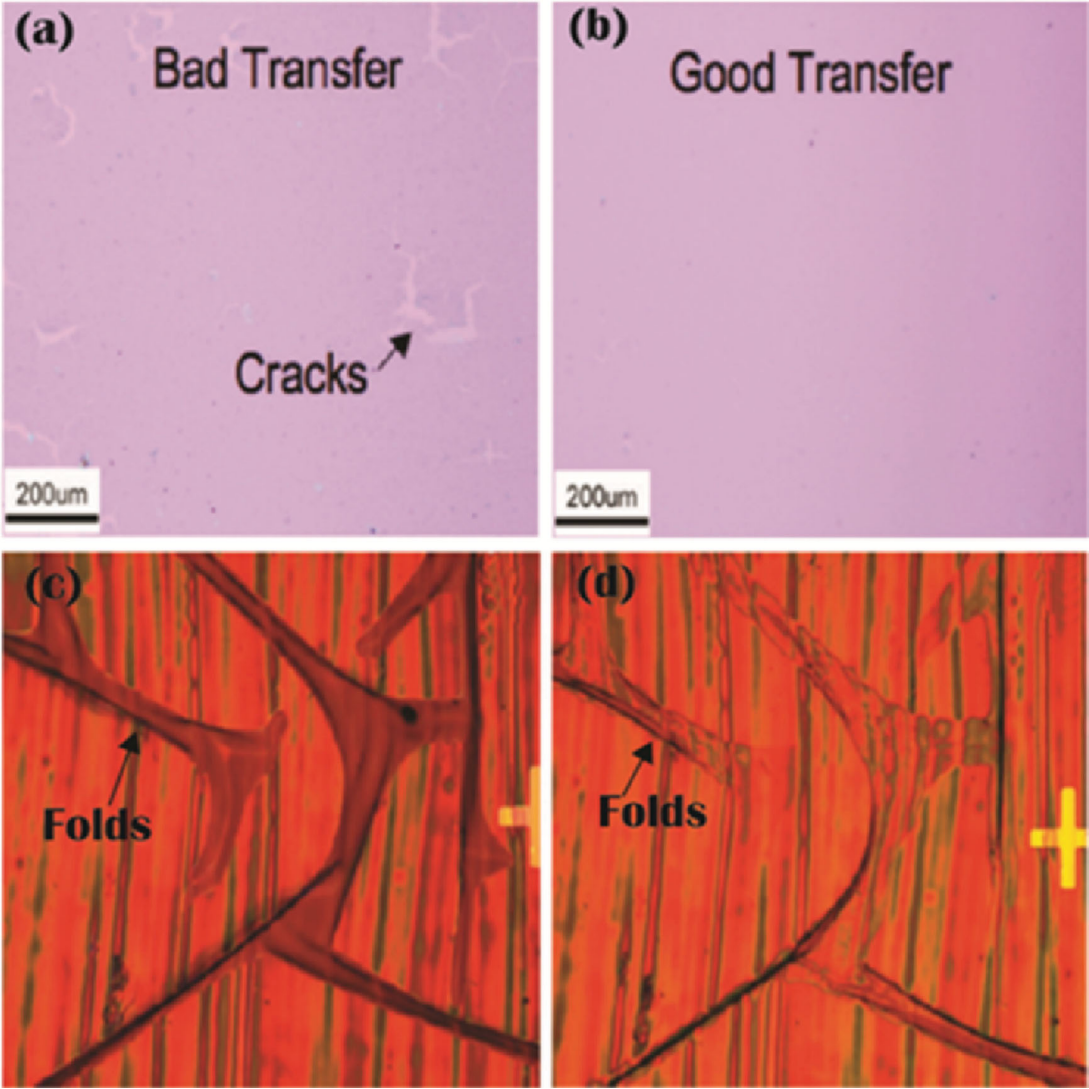
Optical micrographs of graphene transferred on the SiO_2_/Si wafers by a) the traditional method and b) second coated PMMA method. Optical images of large folds c) before and d) after baking the stack at 150 °C. a,b) Reproduced with permission.^41^ 2009, American Chemical Society. c,d) Reproduced with permission.^42^ 2011, American Chemical Society.

In order to make the graphene to be better contacted with the substrate, Li and co‐workers developed an improved method by adding a second PMMA coating after the PMMA/graphene was placed on the SiO_2_/Si substrate.^41^ After Cu was etched away, the PMMA layer was coated again and cured. Then, it was removed by acetone. Graphene transferred by this improved method was of little crack (**Figure** **2**a,b). Liang et al.^42^ found that gaps were also formed when the PMMA/graphene/target substrate stack was dried. After the metal substrate removal, the graphene was placed on the target substrate and dried. A small amount of water may remain in the gaps between the stack and the substrate, which would cause cracks after the PMMA was removed. It is believed that baking the sample before removing the PMMA to evaporate the water between the graphene and PMMA, which could improve the quality of transferred graphene.^42^, ^43^ Liang et al.^42^ baked the sample at 150 °C for 15 min to evaporate the residual water and improve the contact between graphene and the substrate. The surface roughness of the graphene was greatly reduced after baking (Figure 2c,d). They found that this method was more effective in reducing the number of cracks than adding a second step to coat the PMMA layer.

#### 2.1.3 Obtaining Graphene Film with Clean Surface

PMMA layer was removed by acetone in the wet‐transfer method. Due to the high molecular weight and high viscosity of PMMA, it is inevitable to leave PMMA residues on the graphene surface without further annealing. However PMMA residues may trigger charged impurity scattering and unintentional graphene doping thus affect the property of graphene.^44^ For example, the residues can increase the intrinsic sheet resistance (30 Ω sq^−1^).^45^ The high sheet resistance will affect the application of graphene in conductive electrode.^41^, ^46^ Improving the transfer process to achieve the cleanness graphene will be of great importance to fabricate the electronic devices.

**Figure 3:**
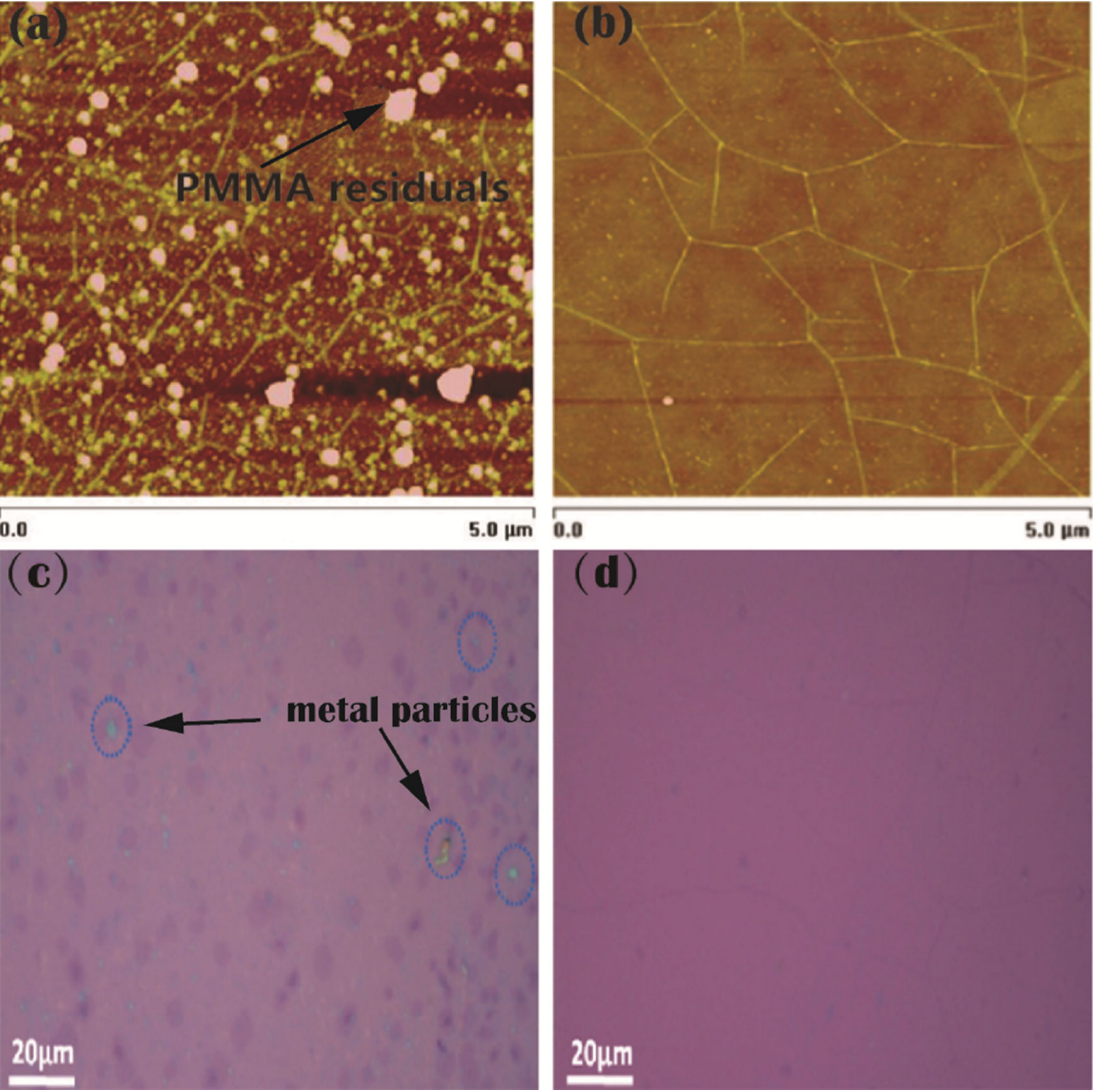
The AFM images of the graphene surface after removal of PMMA via different routes. a) The traditional method: PMMA was treated by acetone for 2 h. b) The improved method: acetone vapor, 2 min acetone immersion, and 3 h of annealing. The typical high‐magnification optical images of the graphene film c) before and d) after the “RCA” process. a,b) Reproduced with permission.^49^ 2012, American Chemical Society. c,d) Reproduced with permission.^42^ 2011, American Chemical Society.

It is believed that annealing in high temperature (250 °C–350 °C)^47^, ^48^ is an efficient way to remove the residues on the graphene surface. However, these methods are unavailable to flexible substrates because of the high annealing temperature. Park et al.^49^ also considered several methods to remove the PMMA residues. For one of these methods, the sample was dealt with acetone vapor, and then was dipped in acetone for 2 min, finally was followed by annealing for 3 h. This method seemed to be the best way to achieve the cleanest surface of graphene (**Figure** **3**a,b).

For the above improved methods, the subsequent processes were added to remove the PMMA residues. However, the adding steps will make the manipulation more complex, which may introduce defects to the graphene and increase the cost. Many simple improved methods have been reported to reduce the PMMA residues. Taking advantage of the excellent solubility of the organic small molecular in the solvent, Han et al. inserted an organic small molecular buffer layer between the PMMA and graphene.^50^ This method is an efficient way to reduce the PMMA residues on graphene due to the good solubility of buffer layer in the solvent. Besides, UV irradiation is beneficial to the degradation of PMMA. The degraded PMMA can be dissolved easily in acetone. Jeong et al.^51^ used the UV irradiation to degrade the pre‐coated PMMA, then coated the PMMA again. The PMMA layer was removed by the mixed solvent of isopropyl alcohol (IPA), acetone, and methyl isobutyl ketone (MIBK). Graphene transferred by this method almost had no apparent polymer residue on the surface.

Scientists also tried to look for the suitable replacer of PMMA layer to get the clean and doping‐free transfer of LAG graphene. Kim et al.^52^ used a pentacene (C_22_H_14_) thin film as the supporting layer had transferred the graphene to 6 in. Si wafer with clean surface and almost no crack. The 200 nm thick pentacene was thermally evaporated onto CVD‐grown graphene on a copper foil to be used as a supporting layer. After transferring the pentacene/graphene onto a target substrate, pentacene was removed by thermal evaporation (250 °C and 300 °C for 1 h) and chemical solvent (tetrahydrofuran (THF)). They also tried other solvent such as benzene, toluene, and chlorobenzene (CB). As a result, the THF was believed to be the best solvent for removing pentacene layer. The graphene transferred by this method showed excellent electrical properties with the hole and electron mobility observed in FETs were 8050 and 9940 cm^2^ V^−1^ s^−1^, respectively. These values were almost two times higher than the field‐effect mobility of the graphene transferred by the PMMA‐assisted method.

**Figure 4:**
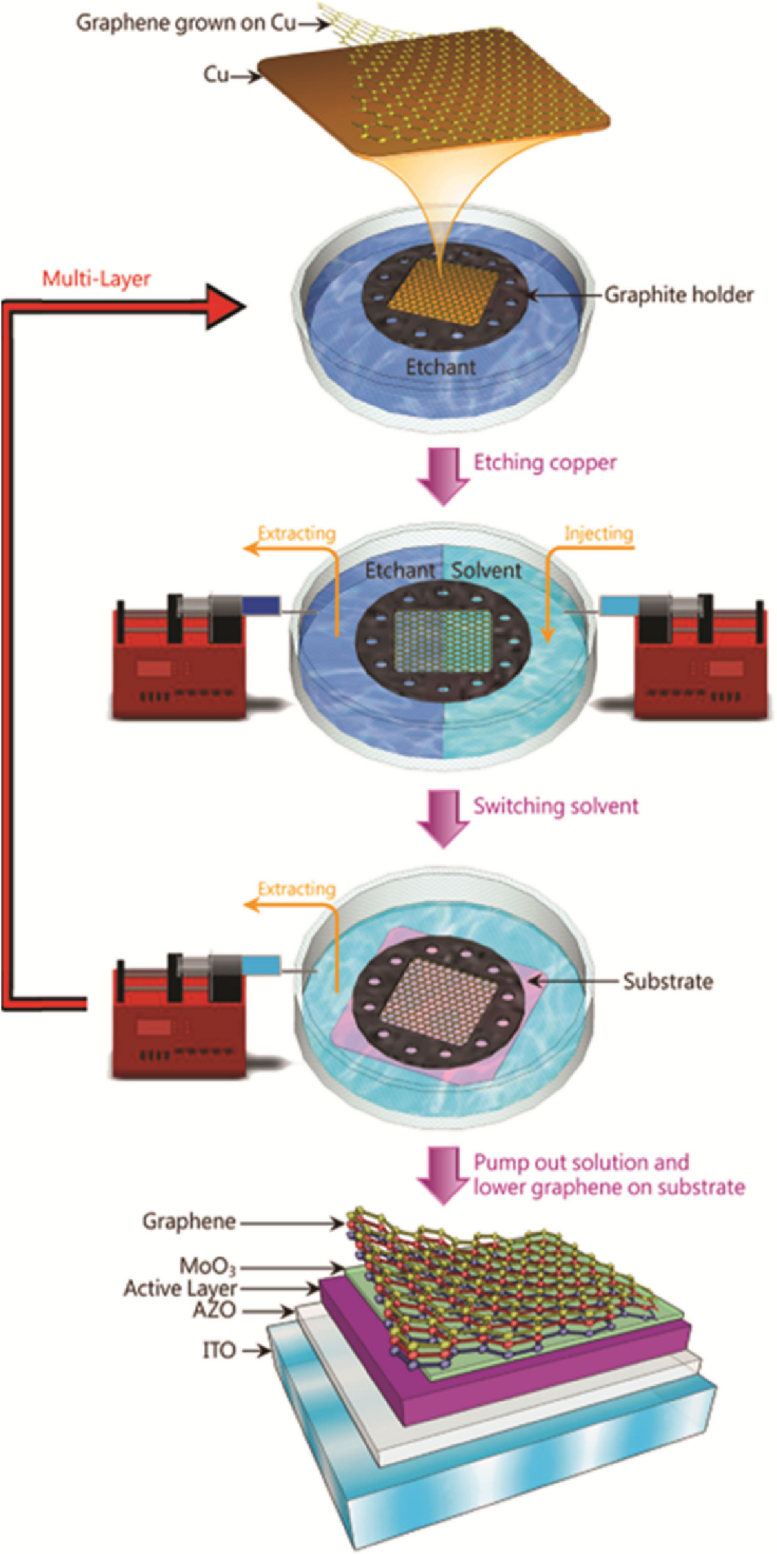
Schematic diagram of the “Polymer‐free” transfer method. Reproduced with permission.^54^ 2014, American Chemical Society.

Transferring graphene without using the polymer supporting layer may be the best way to reduce the polymer residues. These kinds of methods are called the “polymer‐free” methods.^53^ Lin et al. transferred the graphene to the target substrates in a “polymer‐free” way by using a graphite holder (**Figure** **4**).^54^ They used a thin graphite holder as a confinement area for the monolayer graphene. After the Cu substrate was etched, the etchant was pumped at a rate of 0.3 mL min^−1^ and the mixed water/IPA solution was simultaneously injected at the same rate. The surface tension for graphene in the solution could be controlled by this way. The solution was pulled out with the syringe to lower the graphene onto the substrate when the etchant was replaced by the mixed solvents. Compared with the graphene transferred by the conventional polymer‐assisted methods, the graphene transferred by this polymer‐free method showed advanced electrical properties. The mobility of the polymer‐free monolayer graphene is as high as 63 000 cm^2^ V^−1^ s^−1^, which is 50% higher than the similar graphene transferred by the conventional method. But the shape and size of the transferred graphene films are limited by the graphite holder.

To transfer graphene with unrestricted shape, Pasternak and co‐workers used a waterproof marker‐frame to transfer graphene by “polymer‐free” method.^55^ The marker frame was placed on the graphene surface to prevent graphene from cracking and drowning during the transfer process. So the size of the transferred graphene will not be limited in principle. When Cu was completely etched by ammonium persulfate, the graphene was rinsed by DI water. Finally, the water was removed by a tap and graphene fell onto the target substrate.

Wang et al. developed a unique technique called the “clean‐lifting transfer” method by using the electrostatic force to transfer graphene onto target substrates.^56^ The mechanism of this method was using an electrostatic generator to induce the discharge process between the electrostatic generator and the substrate (SiO_2_/Si or PET). The negative charges formed on the target substrates. The graphene was thus attracted to the target substrate by the electrostatic force during the transfer process. After the metal substrate removal, graphene was transferred onto the target substrate. Graphene can be transferred layer by layer in this way. These methods did not use the polymer supporting layer, which enabled the clean transfer of CVD‐grown graphene.

The “polymer‐free” methods that direct transfer the graphene from the growth substrate to the target substrate provide a way to transfer graphene without polymer residues. The interfacial bonding condition between the graphene and the target substrate is critical for transfer success when direct transfer the graphene film to the soft substrate. A key factor for high quality graphene transfer is the wettability of the target substrate. Martins et al.^57^ found that if the substrate was hydrophilic the etchant could enter the interface between graphene/copper stack and the target substrate, so a well‐contacted graphene/copper stack and the soft substrate might become detached during the etching process. Du et al.^58^ presented a theoretical model to derive the wettability requirements of the soft substrate to sustain the direct transfer of graphene, and verified the theoretical analysis with experiments. They used the PDMS as the target substrates, and treated them by oxygen plasma with different time to obtain different degrees of wettability. The experiment results verified that the surface energy components of the substrate had a crucial effect upon the graphene transfer, and that substrates possessing a strong polar surface energy were not suitable for transfer. They found that the critical water contact angle (WCA) of the soft PDMS substrate that can guarantee the direct transfer of graphene lies between 58° and 63°, which was close to the theoretical prediction of about 50°. These results provide guidelines for choosing proper substrates to direct transfer graphene with high quality.

Etchants are efficient to remove the copper substrate, and the commonly used etchants are aqueous solutions of iron nitrate,^41^ iron chloride,^43^ and ammonium persulfate.^28^ After the removal of metal substrate, the metal particulates were found to remain on the graphene surface in the scanning electron microscope images. These metal particulates can hardly been washed off by the washing process. They tend to act as scattering centers to degrade the carrier transport properties and subsequently device performance of graphene.^42^ So, it is necessary to remove these metal particulates.

A cleaning technology called “RCA cleans” is used for Si wafer cleaning in the semiconductor manufacturing. The traditional RCA cleans involve three sequential steps: (1) the first step is intended for the removal of insoluble organic contaminants with a H_2_O/H_2_O_2_/NH_4_OH (5:1:1) solution; (2) the second step is oxide strip using a diluted H_2_O/HF (50:1) solution to remove a thin silicon dioxide layer where metallic contaminants may have accumulated as a result of step 1; (3) in this step, ionic and heavy metal atomic contaminants are removed by using a solution of H_2_O/H_2_O_2_/HCl (5:1:1).^42^ The “modified RCA clean” method has been used for graphene transfer to remove the metal particulates. Liang et al. only adapted both step 1 and step 3 for cleaning the graphene films by diluting the solutions to 20:1:1 and operating at room temperature, and step 3 was performed firstly.^42^ They found reversing the cleaning steps in this manner yielded superior results. Almost no metal particulate was found on the graphene surface after the RCA process (Figure 4c,d).

#### 2.1.4 Electrochemical Delamination Methods

The traditional wet transfer methods that use the etchants to etch the metal substrates have many disadvantages, such as time‐consuming, high cost (because the metal substrates cannot be reused), the metal particulates residues and serious environmental pollution. These disadvantages will no doubt restrict the industrial scalability application of the wet transfer methods.

**Figure 5:**
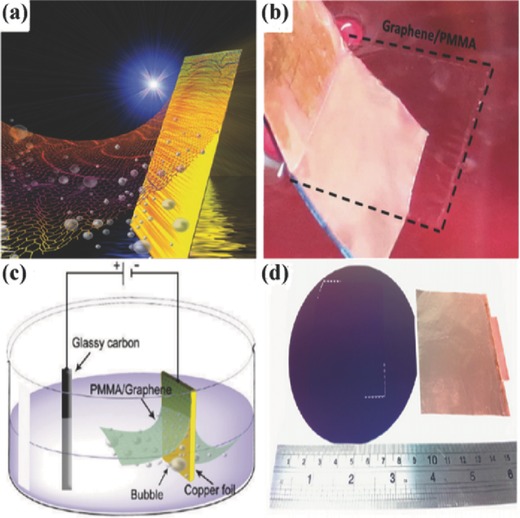
a) Illustration of the “bubble” transfer method. b) Illustration of the “bubble‐free” transfer method. c) Schematic diagram of the electrochemical exfoliation method. d) Graphene was transferred onto a 4 inch wafer by the “bubble” transfer method. a,c,d) Reproduced with permission.^59^ 2011, American Chemical Society. b) Reproduced with permission.^62^

The electrochemical delamination method^59^ (**Figure** **5**a,c,d) can realize the nondestructive transfer of the graphene grown on both sides of the metal substrates. It is very efficient and the metal substrate can be reused. The usually steps are described as follows: (1) PMMA layer is spin‐coated on graphene as a protection layer; (2) taking the PMMA/graphene/metal stack as the cathode electrode, the stack is inserted in the aqueous solution; (3) after applying the voltage, the hydrogen bubbles are emerged at the graphene/metal interface by the electrolysis of water, finally the graphene is delaminated from the edges of the metal substrate; (4) graphene is transferred onto the target substrate after removing the PMMA. The traditional wet transfer methods are not available for the chemically insert or noble metal substrates (Ir, Pt, and Au) because these metals are difficult to etch away completely and this method have a high cost. The electrochemical transfer method can realize the transfer of graphene grown on these substrates.

Gao et al.^60^ used this method to transfer the graphene grown on the Pt substrate with almost nondestructive. The PMMA/graphene/Pt stack served as the cathode electrode in a 1 m NaOH aqueous solution under a constant current of 1 A, with the applied voltage of 5–15 V. Graphene film of 1 × 3 cm^2^ was separated from the Pt substrate in 30 s. What is more, the Pt substrate can be repeatedly used for graphene growth. But when taking the PMMA/graphene/Cu stack as the anode electrode,^61^ the Cu substrate will be oxidized totally. The anode electrode was inserted in the 0.5 m H_2_SO_4_ electrolyte with a potential of 0.5 V and a current of about 0.03 A. It was enough to etch away the Cu foil rapidly. The whole process ended within several minutes, which was much faster than the conventional wet transfer methods. But the Cu substrate cannot be reused.

The electrochemical delamination methods in above^59^, ^60^ can be called “bubble” transfer method because the delamination of the graphene from the metal substrate was induced by the hydrogen bubbles produced by the electrolysis of water. However, the production of hydrogen bubbles during the electrochemical delamination process can cause mechanical damage on the transferred graphene. Cherian et al.^62^ developed a “bubble‐free” transfer method to avoid this problem (Figure 5b). In the “bubble‐free” method, the delamination of graphene from the metal substrate was induced by etching away the oxide metal substrate, which formed by the permeated air. The reduction of copper oxide layer requires potential (−0.8 V), which is lower than that required for hydrogen generating by electrolysis of water (−1.5 V). So no bubble was emerged during the delamination process. The “bubble‐free” transfer method resulted in 0.32 ± 0.2% cracked area, while the “bubble” transfer method resulted in 6.9 ± 5.7% cracked area.^62^ Obviously, the “bubble‐free” method can significantly reduce the defects. Pizzocchero et al.^63^ also demonstrated a non‐destructive electrochemical method to transfer the graphene films with the size up to 100 mm diameter. This was also a “bubble‐free” method proceeding the oxidative and decoupling of copper. The copper/graphene/polymer stack was connected as the working electrode of an electrochemical cell. Hydrogen bubbles were avoided by applying a fixed potential of −0.4 V to the copper/graphene/polymer stack electrode versus an Ag/AgCl reference electrode, which was significantly below the threshold for hydrogen production by electrolysis of water. The oxidation of the copper occurred from the edge of the graphene layer due to the dissolved oxygen in the electrolyte and then towards the center at an average velocity of 24 mm per hour. The applied potential was sufficient to reduce the copper oxide back to metallic copper. This oxidative decoupling process resulted in the delamination of graphene/polymer stack from the copper substrate. The copper substrate could be reused for growth graphene. The growth‐transfer cycle was repeated for a total of five times. Raman D:G peak ratio of the transferred graphene by this method was around 0.1, which was lower than the traditional transfer method with a ratio of 0.26. This result indicated the better quality of the graphene transferred by the “bubble‐free” method.

The wet transfer method is an important way to transfer graphene. However, as we have mentioned above, it has many problems, for example, it is easy to form defects, easy to induce impurity residues on the graphene surface, time consuming, high cost, environment pollution. Etchant and chemical solvents are used in the wet transfer methods. The solvents will introduce the unwanted doping to graphene,^64^ which consequently affect the property of the transferred graphene. These problems restrict the industrial scalability application of the wet transfer methods. So develop the efficient transfer methods that can be applied with industrial scalability will be beneficial to promoting the application of the graphene. The properties of the transferred graphene film by electrochemical delamination methods and “bubble‐free” transfer method are listed in Table 1.

#### 2.1.5 Characterizations of the Graphene Film

**Figure 6:**
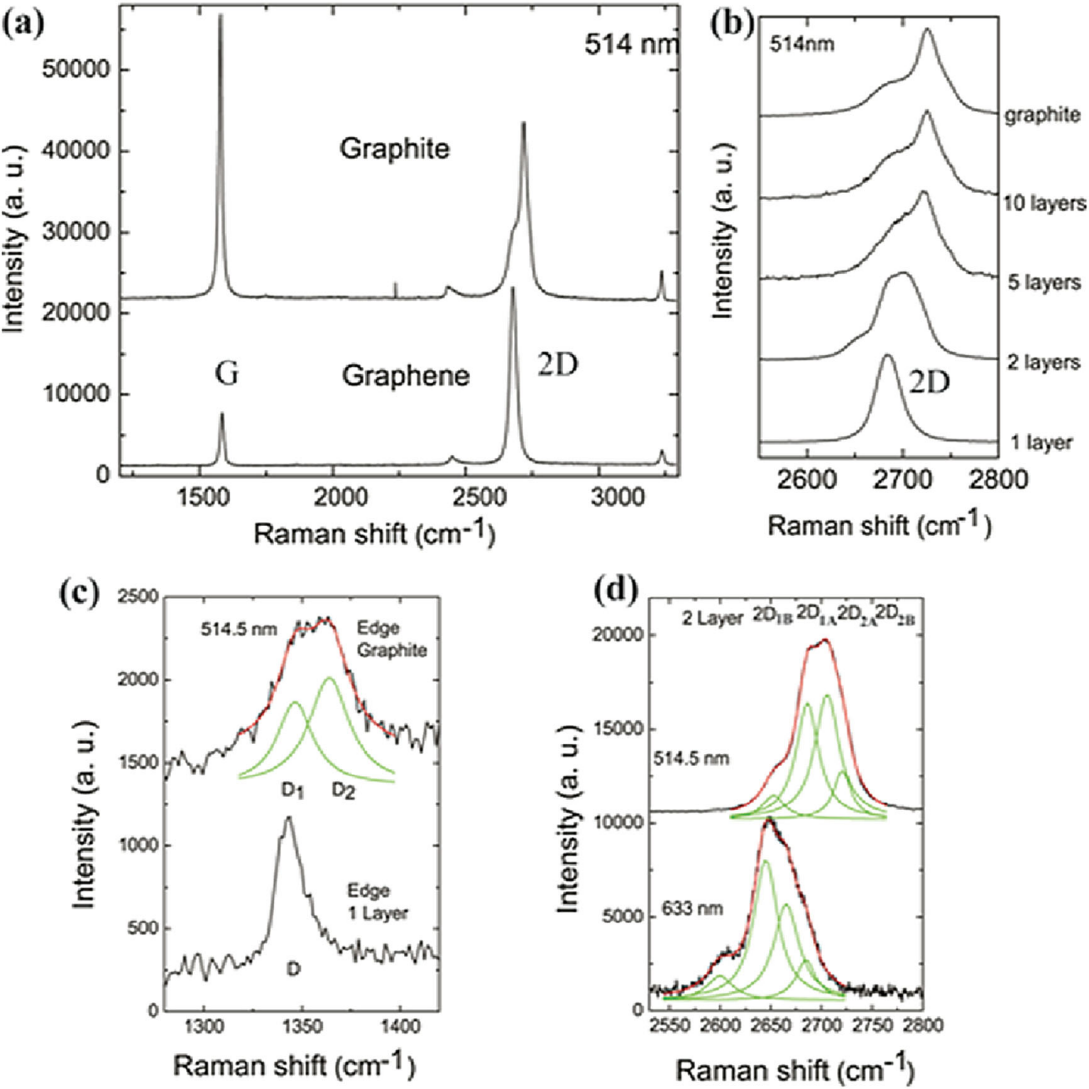
a) Raman spectrum at 514 nm for bulk graphite and graphene. b) Raman spectrum of graphene at 514 nm with the number of layers. c) D peak at the edge of bulk graphite and single layer graphene. d) The four components of the 2D peak in two layer graphene at 514 and 633 nm. Reproduced with permission.^65^ 2006, American Physical Society.

Raman spectroscopy is usually used to characterize the graphene layers, defects, and the doping concentration of graphene. The G peak (≈1580 cm^−1^) and 2D peak (≈2700 cm^−1^) are the typical peaks for graphene (**Figure** **6**a). If there are defects in the graphene or at the edge of the graphene sample, D peak (≈1350 cm^−1^) will emerge in the Raman spectrum (Figure 6c). The quality of graphene can be assessed from the intensity of the D peak. The G peak is related to *E*_2g_ phonon mode at the Brillouin zone center. 2D peak is the second order of zone‐boundary phonons, and D peak is associated with the defects of the crystal.^65^, ^66^ The broad and up‐shifted 2D peak indicates the increase of the graphene layers, as demonstrated in the (Figure 6b). For the bilayer graphene, the 2D peak has four components, while the 2D peak in the single‐layer graphene only has one component (Figure 6d). The 2D band for single‐layer graphene has a full width at half maximum (FWHM) of ≈24 cm^−1^. The intensity ratio of 2D and G peak (*I*_2D_/*I*_G_) can be used to estimate the graphene layers and the doping level of graphene.^67^, ^68^, ^69^ The decrease of the *I*_2D_/*I*_G_ indicates the increase of the graphene layers, while the increase of the *I*_2D_/*I*_G_ usually indicates the lower doping level of graphene. Since D peak is associated with the defects of the graphene film, the lower *I*_D_/*I*_G_ indicates the higher quality of graphene film. So, the values of *I*_2D_/*I*_G_ and *I*_D_/*I*_G_ can give us information of graphene quality. Raman spectroscopy is a very powerful tool to test the quality of graphene film. The development and application of the Raman technology will notably promote the study of the graphene film.

Besides the Raman spectroscopy, the optical micrographs (OM), atomic force microscope (AFM) and scanning electron microscopy (SEM) are also efficient tools to characterize the surface morphology of the transferred graphene. These images can give direct information to determine whether there are contaminants or wrinkles on the graphene film. And AFM can also be used to determine the number of graphene layers from the film thickness. But when taking for practice, it is only possible to distinguish between one and two layers by AFM if the graphene contain folds or wrinkles.^1^

### 2.2 Dry Transfer Method

As we mentioned in the beginning of the section 2, the transfer methods for CVD graphene can be divided into wet and dry transfer methods. For the dry transfer methods, the step of transferring graphene onto the target substrate does not relate to any solutions. Just as the role that PMMA played in the wet transfer method, PDMS^24^ and thermal released tap (TRT)^70^ are often used as the polymer supporting layers during the dry transfer process. These polymer supporting layers can be removed by thermal treatment.

**Figure 7:**
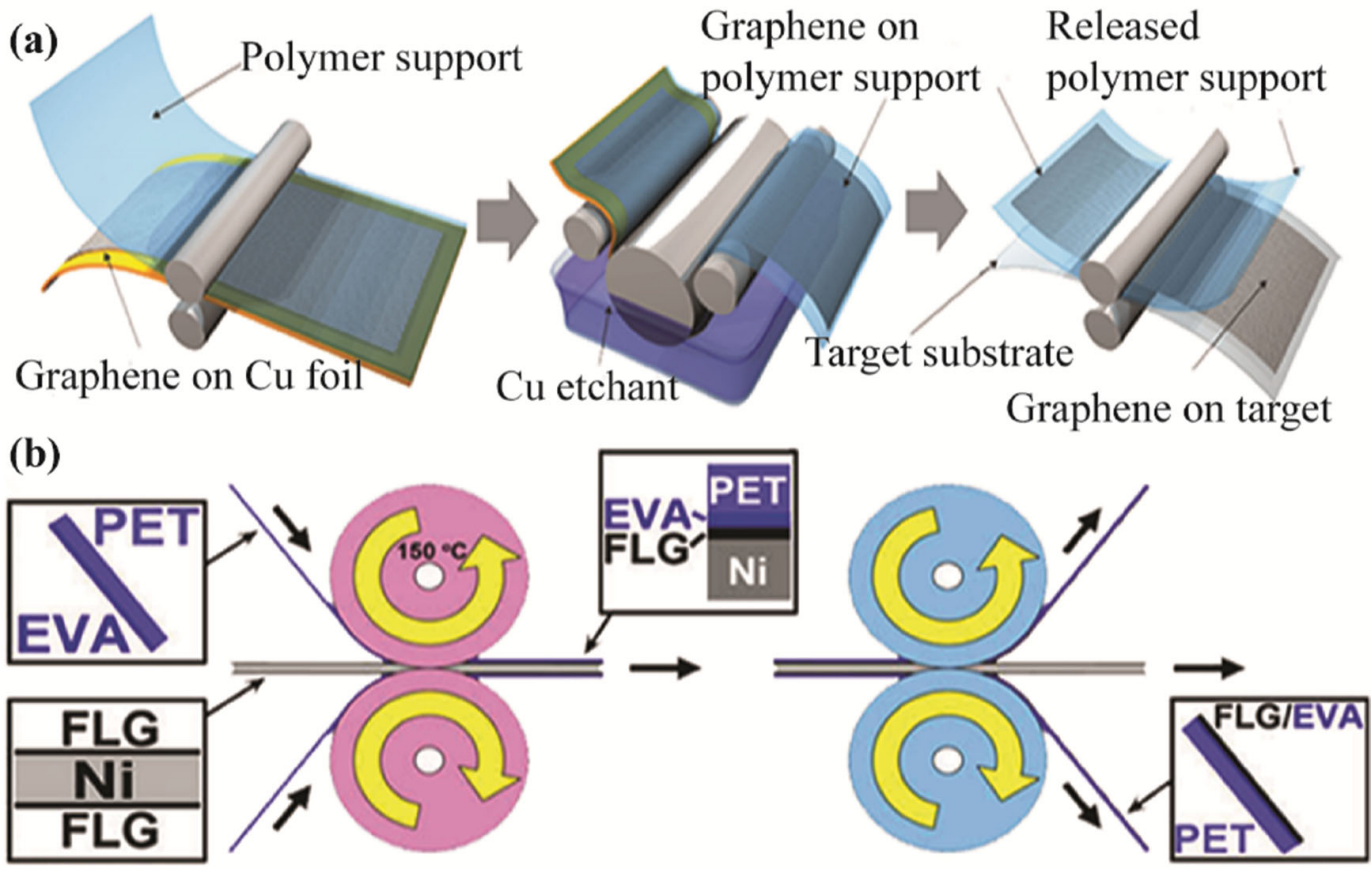
The schematic diagram of the two kinds of “roll‐to‐roll” transfer methods. a) Reproduced with permission.^28^ 2010, Nature Publishing Group. b) Reproduced with permission.^77^ 2010, Elsevier.

The “Roll‐to‐roll” method of synthesizing and transferring the 30 in. graphene has been reported in 2010 (**Figure** **7**a).^28^ First, a TRT was attached to the graphene by two rollers, and then the copper foil was etched away by the copper etchant. Graphene was transferred to the target substrates through the two rollers, and the thermal released tape was simultaneously removed by the mild heating. The 30 in. graphene has been successfully transferred by this method, so this “Roll‐to‐roll” method can be applied to transfer the LAG. Ryu et al.^16^ also used the “Roll‐to‐roll” method to transfer the graphene synthesized by hydrogen‐free rapid thermal chemical vapor deposition. They transferred graphene films over 400 × 300 mm2 to the polyethylene terephthalate (PET) substrate with the sheet resistance of 249 ± 17 Ω sq^−1^ without additional doping. They also fabricated graphene‐based capacitive touch‐screen devices, which demonstrated the potential application of graphene as electrode in the capacitive multitouch devices installed in the most sophisticated mobile phones. (There are two other “Roll‐to‐roll” methods, we will discuss them later.) The adhesion of subsequent graphene and dielectric layers on flexible substrates is very poor, so it is difficult to fabricate graphene‐based flexible devices where more than one graphene layer is need. Bointon et al.^71^ developed a novel cold‐wall CVD method to grow the high quality of monolayer graphene, and demonstrated for the first time a flexible and transparent capacitive touch sensor using graphene for both the top and bottom electrodes. The touch sensor device was fabricated using a novel technique where all lithography was performed on the surface of a CVD graphene covered copper foil. After lithography, the graphene was transferred to a clean polyethylene naphthalate (PEN) substrate by the PMMA‐assisted method. A second set of graphene strips were transferred on top of the PMMA/graphene/PEN. This work demonstrated the graphene touch sensor was suitability for use in next‐generation flexible portable devices.

**Figure 8:**
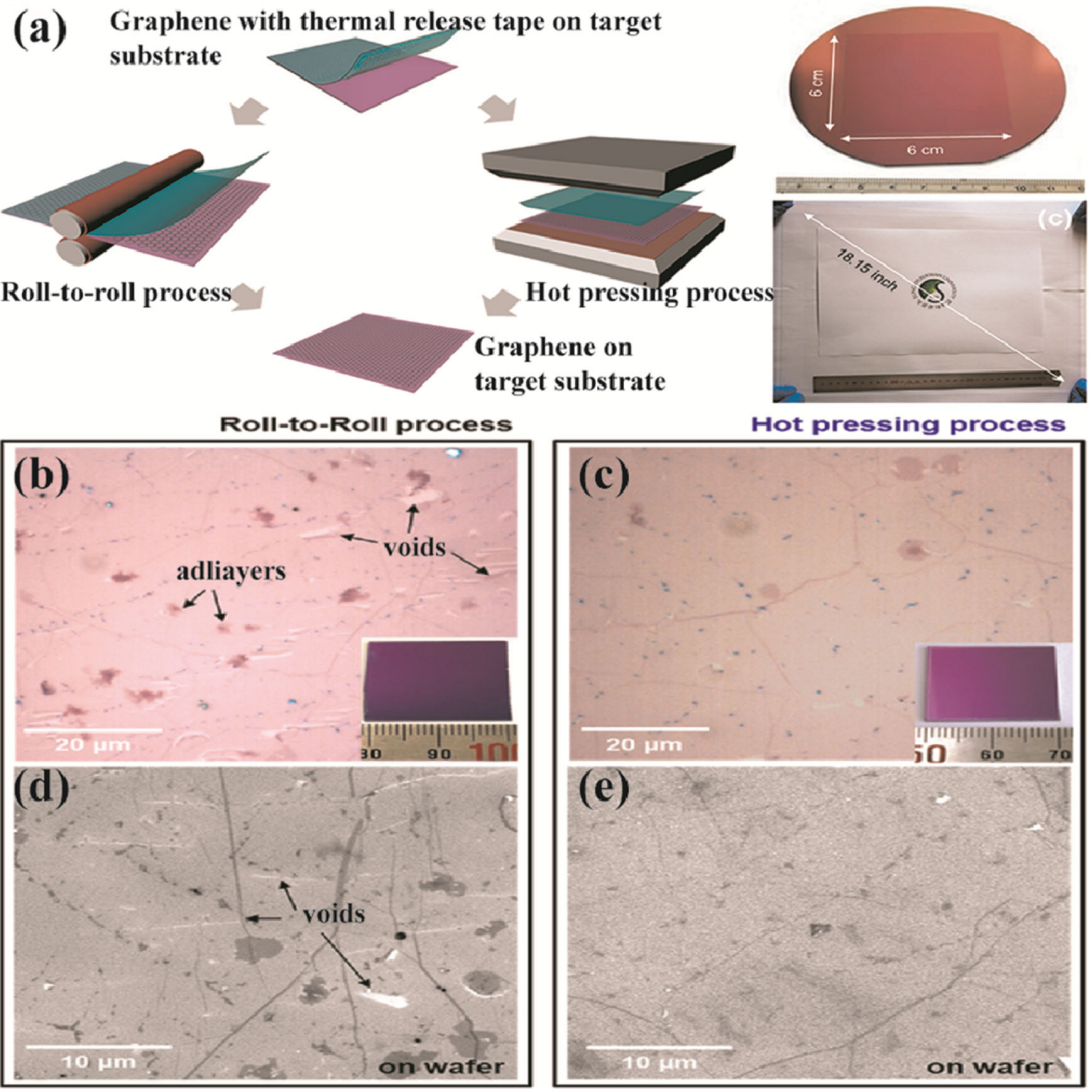
a) Schematic diagram of graphene transferred by “roll‐to‐roll” and hot pressing. b,c) Optical and c,d) SEM images showing the surface morphologies of the graphene films transferred onto SiO_2_/Si substrates by “roll‐to‐roll” and hot pressing respectively. Reproduced with permission.^72^ 2012, American Chemical Society.

The “roll‐to‐roll” method is suitable for the flexible substrates, but mechanical defects are easy to be formed during the transfer process when transfer the graphene to the rigid substrates. Kang et al.^72^ reported an improved method by using two hot pressing plates (**Figure** 8a). The two hot metal plates were applied with controllable temperature (125 °C) and pressure (4 N m^−2^). The TRT was also used as the supporting layer in this process. After the removal of Cu substrate, graphene on the TRT was inserted into two rollers to attach the target substrates (PET or SiO_2_/Si). The TRT lost the adhesion force after the process of hot pressing for less than 10 s, then graphene was transferred onto the target substrate. The improved method can transfer graphene to the flexible or rigid substrates. Compared with “Roll‐to‐roll” method, graphene transferred by the “hot pressing” method forms relatively less defects (Figure 8b–e).

In the two methods above, the TRT are used to support the graphene film by taking advantage of the viscosity of TRT. The adhesion between the polymer supports (TRT) and the graphene is very strong, so it is inevitable to leave adhesive residues on the graphene surface. In order to get a clean surface of the transferred graphene, Chen et al.^27^ took advantage of the dispersive adhesive of the silicon without using any chemical adhesive. The adhesion between the graphene and the silicon was achieved by van der Waals force. Due to the weak adhesion and the low surface tension of silicone, the graphene was transferred onto the target substrate successfully with a cleanness surface. The polymer/silicon can be reused to reduce the cost. So this method is an efficient way to transfer LAG with cleanness surface and high quality.

Kim et al.^73^ used the pressure‐sensitive adhesive films (PSAF) as the supporting polymer layer by taking advantage of the difference in wettability and adhesion energy of graphene with respect to PSAF and a target substrate. The graphene coated with the PSAF was attached to the target substrates by week pressing or rolling methods with normal stress of 4 N mm^−2^ after etching away the copper substrate. The PSAF layer was peeled off from the graphene/target substrate with the speed and angle with respect to substrate surface were 2 mm s^−1^ and about 90°, respectively. The graphene transferred by this method had cleaner surface than transferred by the PMMA and TRT. The PSAF‐assisted transfer method is applicable to both rigid and flexible substrates without difficulty in scaling up for industrial application.

In the dry transfer methods discussed above, the etchants have also been used to remove the Cu substrates, so Cu foil cannot be reused to grow graphene. Graphene is grown on both side of the Cu foil by CVD method. One side of the graphene must be removed in advance before etching away the Cu substrate. This process causes a lot of waste. So there are still many problems that restrict the industrial scalability application of these methods. Zaretski et al.^74^ developed a “Metal‐assisted exfoliation” method. A 150 nm metal film (nickel or cobalt) was deposited on the graphene as the supporting layer then TRT was attached to the metal layer. Owing to the different adhesion of graphene to various metals, the metal/graphene was exfoliated from the Cu substrate when peeling up the TRT. Finally, TRT and metal layer were removed by heating and chemical etchants, respectively. This method is also a “Roll‐to‐roll” process. Both sides of the graphene grown on Cu substrate can be transferred and the Cu foil can be reused. But etchant was also used to remove the metal‐assisted layer, and unwanted doping was introduced into graphene by the chemical solvent,^64^ which would affect the property of the transferred graphene.

To realize the real dry transfer process, the adhesion energy between the graphene and substrate should be measured for the precise control of the delamination process. Yoon et al.^75^ measured the adhesion energy by the double cantilever beam fracture mechanics testing. The adhesion energy between the graphene and the substrate used in the study was found to be 0.72 ± 0.07 J m^−2^. And they used the dry transfer method to transfer the graphene to the polyimide (PI) by the assistance of an epoxy layer. The Cu etchant was avoided in this process, and Cu substrate can be reused to grow graphene.

It is very important to achieve highly conformal contact between the graphene and the target substrate to obtain high adhesion energy between them. Jung et al. developed a mechano‐electro‐thermal method,^76^ the strong contact between the graphene and the target substrate was achieved by the assistance of the mechanical pressing as well as the electrostatic force under moderate thermal heating. So the monolayer graphene of 7 × 7 cm^2^ was transferred to the target substrate in short time. Juang et al.^77^ used the “Roll‐to‐roll” (Figure 7b) method to transfer the graphene grown on Ni substrate to the PET substrate. The ethylene‐vinyl acetate copolymer (EVA) acted as the adhesion layer between the graphene and the target substrate. Successively passed the hot rollers and the cold rollers, the graphene was transferred from the Ni substrate to the PET substrate successfully. No chemical etchant was used during this process, so the real dry transfer methods have been achieved. The “Roll‐to‐roll” method may be available for industrial application. This method is suitable for transferring the graphene to flexible substrate, but it may be unavailable for the rigid substrate.

**Figure 9:**
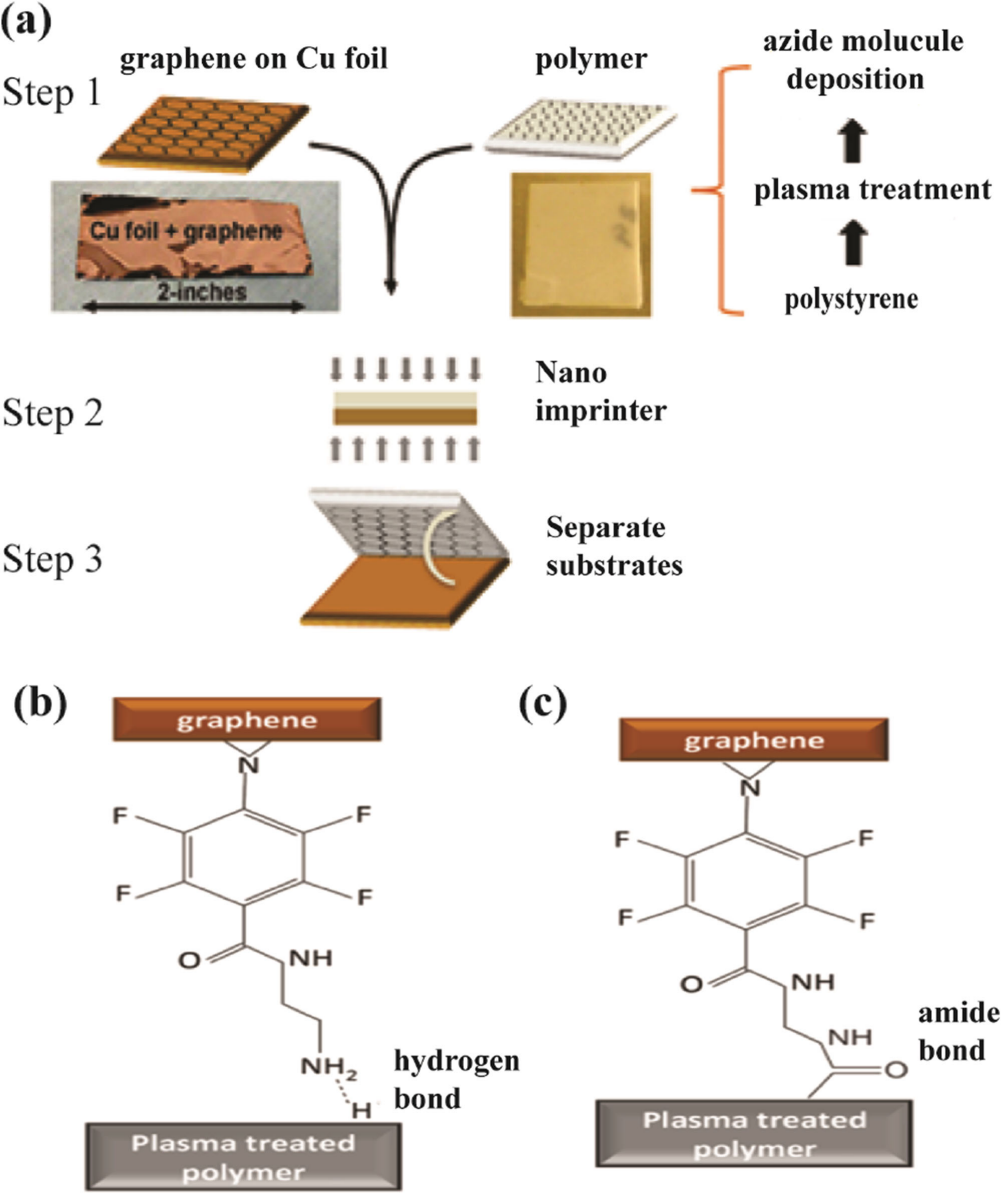
a) Schematic diagram of the dry transfer methods. b) Hydrogen bond and c) covalent bond formed between the TFPA and the polystyrene surface. Reproduced with permission.^78^ 2012, American Chemical Society.

Besides using the adhesives as the above methods done, introducing covalent bonds between the graphene and the substrates has also been adopted to increase the adhesion energy. Graphene can be transferred with high quality without using any chemical etchant. Lock et al.^78^ used an azide as linker molecule to form hydrogen bond or covalent bond between graphene and the target substrate (polystyrene) (**Figure** **9**). The linker molecule *N*‐ethylamino‐4‐azidotetrafluorobenzoate (TFPA‐NH_2_) was deposited on the polymer surface after plasma treatment. This process made the adhesion between the graphene and polystyrene to be much higher than that between the graphene and metal substrate. Graphene can be separated from the metal substrate by the assistance of the nanoimprinter. The whole process took less than 3 h. This method is also suitable for the industrial scalability application. The properties of the transferred graphene film by dry transfer methods are listed in Table 1.

**Figure 10:**
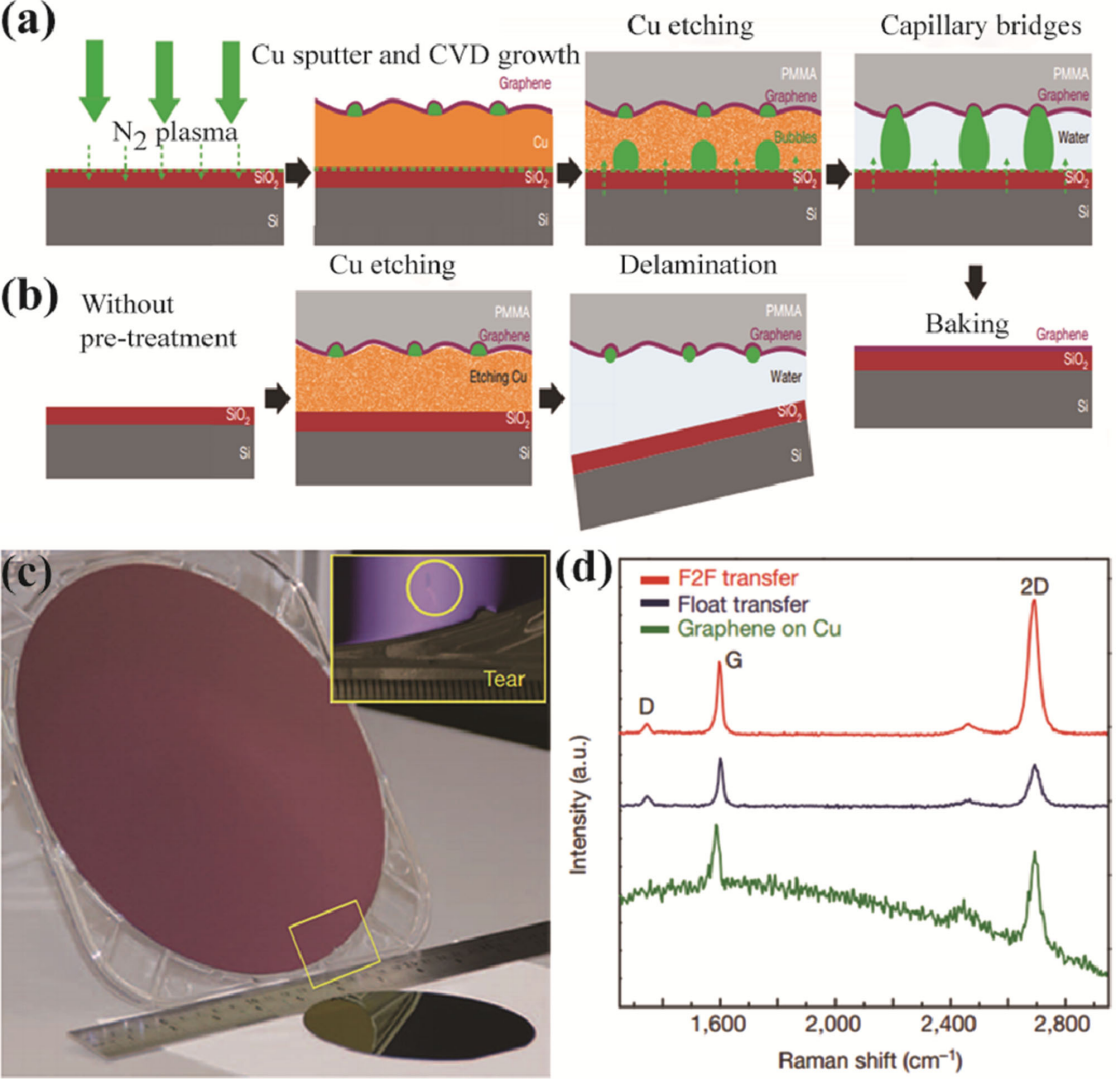
a,b) Schematic diagram of the face‐to‐face method for transferring graphene mediated by capillary bridges. c) Photograph of the transferred graphene on an 8 in. wafer and 4 in. wafer. d) Raman spectrum of graphene transferred by face‐to‐face transfer (red), float transfer onto SiO_2_/Si substrates (blue) and graphene on Cu film before transfer (green). Reproduced with permission.^36^ 2013, Nature Publishing Group.

In above, we review the wet transfer and dry transfer method of graphene grown on metal substrates. Since the “Roll‐to‐roll” dry transfer method has succeeded transferring the 30 in. graphene, it seems to be a more available way for industrial application. But many defects are emerged after transferring. And the disadvantages we have mentioned above suggest that the wet transfer method is difficult to scale up. Gao et al. developed a “Face‐to‐face” transfer method (**Figure** **10**).^36^ During the etching of the Cu foil, the evolution of bubbles leaded to the formation of capillary bridges between the graphene and substrate. So the graphene film remained attached to the substrate during the etching process. The SiO_2_/Si substrate was pretreated by N_2_ plasma to convert the surface of the substrate to silicon oxynitride phases. This step was demonstrated to be helpful for facilitating the formation of capillary bridges. The “Face‐to‐face” method realized both the growth and transfer of graphene on one wafer, and this process did not have to be done by hand, which were different from the wet and dry transfer methods. The quality of the transferred graphene was better than the conventional transfer method.

## 3 The Transfer of Epitaxial Graphene Grown on SiC

We have discussed the transfer methods of graphene grown on the metal substrates by CVD method in the part 2. CVD method is believed to be an efficient way to synthesis LAG. But the surface of the Cu substrate is rough, so graphene grown on Cu substrate is easy to form wrinkles. Besides, the orientation of the graphene cannot be controlled easily. Epitaxial growth of graphene on SiC surface is also an important way to synthesis LAG. High‐quality graphene can be grown on SiC wafer by self‐limiting sublimation of Si. And the synthesized graphene has a single orientation because of the self‐limiting growth process. SiC substrate is insulated, so in some cases it can be used directly without transfer. But the cost of SiC is very high and sometimes the graphene is needed to be transferred to other substrates. It is also necessary to develop the method for transferring graphene grown on SiC wafer. However, SiC is resisted to the chemical etchants. The traditional wet transfer method is not available here.

Graphene grown on SiC substrate can be transferred onto SiO_2_/Si substrate by the assistance of the adhesive tape. But the size of the transferred graphene was limited to be about 1 μm^2^ or less.^79^ This method is not suitable to transfer LAG grown on SiC substrate. The TRT is believed to be a good material to transfer the epitaxial graphene.^80^ The main reason that graphene can be successfully transferred by this method is that the adhesion force between the tape and the graphene is higher than that between graphene and SiC. TRT can be removed by the high temperature, but usually it cannot be removed completely. We have mentioned in part 2.1.1 that the polymer residues on the surface of graphene will affect the quality of graphene. The chemical solvent is used to remove the residues. But it will introduce the unwanted doping to graphene by the chemical solvent,^64^ and affect the property of the transferred graphene. Furthermore, graphene grown on the SiC is usually not a single layer. Multilayer graphene can be transferred by this method, but it is difficult to get the single‐layer graphene by this method.

**Figure 11:**
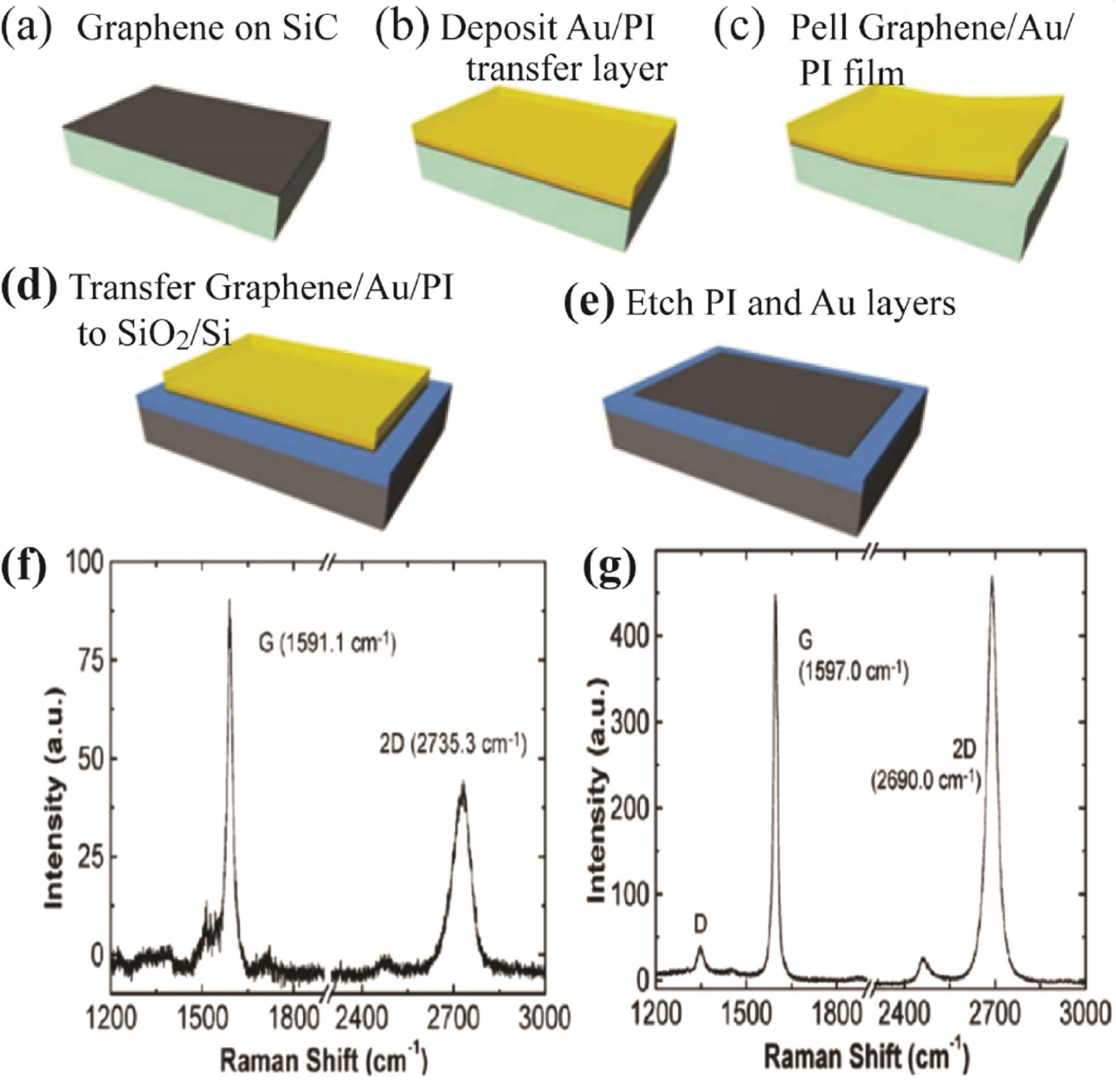
a–e) Schematic diagram of the method to transfer epitaxial graphene grown on SiC substrate. f) Raman spectrum of graphene grown SiC, the width of the 2D peak (72.6 cm^–1^) suggests multilayer graphene. The D peak is not observed in this sample. g) Raman spectrum of the transferred graphene, the D peak is observed in this sample, which suggests a high density of defects of the graphene. a–e) Reproduced with permission.^82^ Copyright 2009, American Institute of Physics. f,g) Reproduced with permission.^83^ 2010, American Chemical Society.

The physical transfer method that has been successfully used to transfer the carbon nanotubes was also adopted to transfer graphene. For this method, the deposited metal layer provides the adhesion force, and a thin polymer layer serves as a supporting layer for the delamination process.^81^ Unarunotai et al., respectively, used the Au/PI layers^82^ (**Figure** **11**a–e) and Pd/PI layers^83^ to transfer graphene from SiC to SiO_2_/Si substrate successfully. The PI and the metal layer were removed by O_2_ plasma reactive ion etching and chemical etchant (Transene), respectively. This method can transfer the graphene film layer by layer. It is also suitable to transfer LAG. But the results of Raman spectrum showed that more defects were formed after transferring (Figure 11f,g).

The multilayer graphene can be transferred through these methods, which mainly depends on the weak interaction between graphene and graphene. The binding energies per pair of atoms between the graphene and metal layers (γAu‐G ≈ 60 meV, γPd‐G ≈ 70 meV) have been estimated by Hamada and Otani.^84^ These binding energies are higher than that between graphene and graphene (γG‐G ≈ 40–50 meV).^85^ So, the graphene can be transferred successfully. But the binding energy between SiC and graphene has been estimated to be 106.2 meV (γG‐SiC ≈ 106.2 meV).^86^ It is larger than the binding energy between Au and graphene as well as that between Pd and graphene. So these two metal layers (Au and Pd) are not suitable to transfer the monolayer graphene grown on SiC. The binding energy between Ni and graphene has been found to be about 140 meV by Hamada and Otani. It is larger than γG‐SiC. So using the Ni as the metal layer can transfer the graphene from SiC directly. Graphene grown on SiC substrate is usually to be more than one layer. The Au layer was used to transfer the transferred graphene by second time to remove the residue graphene on the monolayer graphene. After the two steps transfer, 99% area of the graphene was single layer.^76^

The metal layers have been used in the above transfer methods. When the graphene was transferred to the target substrates, chemical etchants were used to remove the metal layer. Defects were introduced during the etching process easily. The residue metal particles and the doping of graphene introduced by the chemical etchants will influence the property of graphene. Tanabe et al.^87^ used the water‐soluble polymer poly(vinyl alcohol) (PVA) to avoid using the chemical solvent. This method can be called the “Etch‐free” method. Using the adhesion force provided by the PVA, the graphene was transferred to SiO_2_/Si substrate and then the PVA was dissolved by water. No chemical solvent was used during this process. The doping‐free graphene can be obtained by this method.

In above, we have reviewed the transfer methods of the epitaxial graphene grown on SiC substrate. The “Etch‐free” transfer method seems to be an efficient method because it does not use any chemical solvent. This method is a more environment friendly and economical way to transfer the graphene for applications. The dry transfer method^80^ can also be used to transfer graphene grown on SiC substrate. But many issues were occurred by these methods, such as residues and defects. These methods should be optimized to get graphene of large‐area and high quality. However, all of these methods have made it possible to transfer graphene grown on SiC wafer. The development of the transfer methods will promote the application of epitaxial graphene grown on SiC.

## 4 Summary and Outlook

The transfer of graphene acts as the bridge between the production and application of graphene. In this work, we have reviewed the transfer methods of graphene grown on metal (mainly on Cu) and SiC substrates. All of these methods are aimed at transferring graphene with clean surface and little defect. Many transfer methods have achieved these goals. The success of the graphene transfer is of great significance for the application of graphene film, making it possible for the industrial scalability application of graphene. The “Roll‐to‐roll” dry transfer method can transfer the graphene film as large as 30 in., so it seems to be the efficient method for industrial application. Other methods also have their own advantages. Some of them are hopeful for the practical industrial application. Despite that, the transfer methods are still need to be optimized to transfer graphene with little defect and large area. There are still many challenges to transfer LAG films. (1) The first challenge is avoiding the crack during the transfer process while achieve large area transfer at the same time. Monolayer graphene itself can be easily cracked during the transfer process without the assisted of the supporting layer. The improper operation during the transfer process can also induce cracks on the graphene. (2) The second challenge is obtaining LAG film with clean surface. The contaminants on the graphene surface are also big problems to be solved because they may act as scattering centers and induce unintentional graphene doping thus affect the graphene property. It is necessary to obtain LAG film with clean surface when fabricate graphene‐based devices. (3) Simplifying the transfer process to reduce the cost is also a main challenge. The cost of the transfer process must be taken into consideration in the practical industrial application. The complex and time‐consuming transfer methods are unsuitable for transferring the LAG film. Besides, both the transfer of graphene and the synthesis methods of graphene are very important for the application of graphene. It is necessary to optimize the methods to product LAG films with high quality. In recent years, the transfer‐free growth methods where graphene grew directly on target substrates have been reported.^88^, ^89^, ^90^, ^91^ Graphene grown by the “transfer‐free” method can be directly used without further transfer. If these methods can be applied for industrial scalability production of graphene, it will greatly promote the application of graphene. But before these methods become mature, the transfer process still plays a very important role in the application of graphene.
